# Quantification of m6A RNA methylation modulators pattern was a potential biomarker for prognosis and associated with tumor immune microenvironment of pancreatic adenocarcinoma

**DOI:** 10.1186/s12885-021-08550-9

**Published:** 2021-07-31

**Authors:** Lianzi Wang, Shubing Zhang, Huimin Li, Yang Xu, Qiang Wu, Jilong Shen, Tao Li, Yuanhong Xu

**Affiliations:** 1 Department of Clinical Laboratory, the First Affiliated Hospital of Anhui Medical University, No. 218 Jixi Road, Hefei, 230032 Anhui China; 2 Department of Pathology, the First Affiliated Hospital of Anhui Medical University, No. 218 Jixi Road, Hefei, 230032 Anhui China; 3 The Key Laboratory of Microbiology and Parasitology of Anhui Province, the Key Laboratory of Zoonoses of High Institutions in Anhui, Anhui Medical University, Hefei, China

**Keywords:** Pancreatic adenocarcinoma, m6A RNA methylation modulators, m6Ascore, Prognostic, Immune cell infiltration, Immunotherapy

## Abstract

**Background:**

m6A is the most prevalent and abundant form of mRNA modifications and is closely related to tumor proliferation, differentiation, and tumorigenesis. In this study, we try to conduct an effective prediction model to investigated the function of m6A RNA methylation modulators in pancreatic adenocarcinoma and estimated the potential association between m6A RNA methylation modulators and tumor microenvironment infiltration for optimization of treatment.

**Methods:**

Expression of 28 m6A RNA methylation modulators and clinical data of patients with pancreatic adenocarcinoma and normal samples were obtained from TCGA and GTEx database. Differences in the expression of 28 m6A RNA methylation modulators between tumour (*n* = 40) and healthy (*n* = 167) samples were compared by Wilcoxon test. LASSO Cox regression was used to select m6A RNA methylation modulators to analyze the relationship between expression and clinical characteristics by univariate and multivariate regression. A risk score prognosis model was conducted based on the expression of select m6A RNA methylation modulators. Bioinformatics analysis was used to explore the association between the m6Ascore and the composition of infiltrating immune cells between high and low m6Ascore group by CIBERSORT algorithm. Evaluation of m6Ascore for immunotherapy was analyzed via the IPS and three immunotherapy cohort. Besides, the biological signaling pathways of the m6A RNA methylation modulators were examined by gene set enrichment analysis (GSEA).

**Results:**

Expression of 28 m6A RNA methylation modulators were upregulated in patients with PAAD except for MTEEL3. An m6Ascore prognosis model was established, including KIAA1429, IGF2BP2, IGF2BP3, METTL3, EIF3H and LRPPRC was used to predict the prognosis of patients with PAAD, the high risk score was an independent prognostic indicator for pancreatic adenocarcinoma, and a high risk score presented a lower overall survival. In addition, m6Ascore was related with the immune cell infiltration of PAAD. Patients with a high m6Ascore had lower infiltration of Tregs and CD8^+^T cells but a higher resting CD4^+^ T infiltration. Patients with a low m6Ascore displayed a low abundance of *PD-1, CTLA-4 and TIGIT*, however, the IPS showed no difference between the two groups. The m6Ascore applied in three immunotherapy cohort (GSE78220, TCGA-SKCM, and IMvigor210) did not exhibit a good prediction for estimating the patients’ response to immunotherapy, so it may need more researches to figure out whether the m6A modulator prognosis model would benefit the prediction of pancreatic patients’ response to immunotherapy.

**Conclusion:**

Modulators involved in m6A RNA methylation were associated with the development of pancreatic cancer. An m6Ascore based on the expression of IGF2BP2, IGF2BP3, KIAA1429, METTL3, EIF3H and LRPPRC is proposed as an indicator of TME status and is instrumental in predicting the prognosis of pancreatic cancer patients.

**Supplementary Information:**

The online version contains supplementary material available at 10.1186/s12885-021-08550-9.

## Background

Pancreatic cancer is an aggressive cancer of the digestive system with high morbidity and mortality. It is the seventh leading cause of cancer death in both males and females worldwide because of its poor prognosis. The high death rate of pancreatic cancer has been a continuous challenge to the medical field [1]. Data from the National Centre for Health Statistics (NCSH) show a reduction in the occurrence of breast cancer, colorectal cancer, and prostate cancer in the past decade, but the incidence of pancreatic cancer continues to increase, and it has become the fourth leading cause of cancer death [2]. Studies have predicted that it could be the second leading cause of cancer death by 2030. The estimated incidence of pancreatic cancer in the United States in 2020 is around 57,600 with a projected 47,050 deaths, indicating a mortality above 80% [1]. Furthermore, the 5-year survival rate of pancreatic cancer is 9% and is the lowest across all types of cancer. Pancreatic adenocarcinoma (PAAD) is the most common type of pancreatic cancer, which encompasses 85% of pancreatic cancer [3]. While the precise cause and underlying mechanisms of PAAD are still unclear, genetics is an essential factor related to tumour development and mutations in DNA or RNA may also drive the initiation of PAAD [4].

RNA modification is a crucial part of epigenetics, which together with gene and protein modification, plays an important role in regulating cellular processes. According to the data from the MODOMICS update in 2017, about 163 different modifications have been identified in RNA molecules so far, including N1-methyladenosine, N7-methyladenosine, 5-methylcytosine, pseudouridine, N6,2′-O-dimethyladenosine (m6A) and 2′-O-methylation. In eukaryotes, m6A is the most prevalent and abundant form of mRNA modification, which was identified in the 1970s [5, 6]. m6A is a dynamic and reversible process of mRNA modification and has been widely studied, although the exact mechanism of m6A modification remains unelucidated due to the technical limitations. The formation of m6A is composed of three individual proteins, and it is possible to detect these m6A proteins as a reflection of m6A modification. These proteins are classified into three categories: writers, erasers, and readers, which function as modulators in the process of m6A modification. Writer proteins mainly include METTL3, METTL14, WTAP, CBLL1 and METTL16 [7–10]. RBM15, ZC3H13, and KIAA1429 have been newly identified and play a role in mediating the formation of the writer protein complex [11–14]. These writer proteins transfer the methyl group from S-adenosyl methionine to the RNA nucleotides. Erasers, on the other hand, are demethylases and remove the methyl group from the RNA molecule. Members of this group include FTO and ALKBH5 [15, 16]. Finally, reader proteins members that recognise m6A modification, including the family of YT521-B homology (YTH) domain-containing proteins (YTHDF1, YTHDF2, YTHDF3, YTHDC1, and YTHDC2) and the IGF-2 mRNA-binding proteins (IGF2BP1, IGF2BP2, and IGF2BP3) [17, 18], heterogeneous nuclear ribonucleoproteins hnRNPA2B1 and hnRNPC also have been identified as m6A RNA readers [19].

m6A plays a vital role in the splicing, translation, and stability of RNA and regulates chromatin state and transcription [20]. This has significant consequences in various human diseases where m6A alterations may enhance heart failure [21] influence brain development and function [22], immune response to viral infection [23], and bone metabolism [24]. An increasing amount of evidence indicates that m6A RNA methylation modulators are associated with the development and progression of several malignant tumours, and play a dual role in cancer development. On the one hand, m6A regulates the expression of oncogenes or tumor suppressor genes, thereby affecting cancer progression. On the other hand, it can regulate the expression and activity of the m6A enzyme, thereby affecting the role of m6A in cancer. In bladder cancer, m6A modulator METTL3 promotes oncogenes’ expression [25]. Furthermore, YTHDF1 has been linked with EIF3C, leading to the occurrence and metastasis of ovarian cancer [26]. Studies have also found that m6A RNA methylation modulators cause genetic alterations such as mutations and copy number variations across diverse cancer types [27]. Moreover, m6A RNA methylation modulators have been shown to correlate with clinical parameters and have been utilized as tumour biomarkers to determine the diagnosis and prognosis of several cancer types and monitor tumor development [27]. m6A and its modulators are widely studied in hepatocellular cancer [28], colorectal cancer, and genital and nervous system tumours [26, 29–31]. Recently, He et al. demonstrated that ALKBH5 inhibits pancreatic cancer motility by demethylating long non-coding RNA KCNK15-AS1 [32]. Other studies indicate that ALKBH5 decreases WIF-1 RNA methylation and inhibits pancreatic cancer tumorigenesis through the Wnt signaling pathway [33]. However, therapies targeting signaling pathways in pancreatic cancer lack efficacy due to the heterogeneity of tumor microenvironment (TME) in patients, TME prevents antitumor drug resistance, immune escape and tumor cell’s infiltration during cancer progression. Increasing evidence demonstrated that m6A modification is associated with TME of cancer, and the polarization of tumor-associated macrophages-M2 enabled the oxaliplatin resistance via the elevation of METTL3-mediated m6A modification [34]. Dali et al. found a risen level of CD8+ cytotoxic T cells and natural killer (NK) cells while a reduced infiltration of myeloid-derived suppressor cells (MDSC) from Ythdf1−/− tumor mice compared to WT mice, besides, the therapeutic efficacy of PD-L1 checkpoint blockade is enhanced in Ythdf1−/− mice [35].

Since the tumor is a result of the coordinated action of multiple factors and genes, we integrated the gene expression profiles information from The Cancer Genome Atlas (TCGA) and GTEx database, extracted expression of 28 widely reported m6A RNA methylation modulators from 140 patients with PAAD, assessed the influence of m6A RNA methylation modulators in the clinical and pathological characteristics of PAAD comprehensively, explored its relationship with the TME cell-infiltrating characteristics and predicted the efficacy of immune checkpoint inhibitors.

## Materials and methods

### Data collection and pre-processing

RNA-seq transcriptome data (FPKM value) and corresponding clinical information of pancreatic cancer patients were obtained from TCGA database (https://cancergenome.nih.gov/). The gene expression profile was measured experimentally using the Illumina HiSeq2000 RNA Sequencing platform by the University of North Carolina TCGA genome characterization center. We obtained expression profiles of 178 patients with PAAD; however, 34 cases should not have been included in the PAAD study according to the quality annotation from TCGA, and one patient was a repeated measure, furthermore, PAAD patients with incomplete data on age, gender, survival time (futime), survival status (fustat), pathological grade of the tumor, and pathologic stage were excluded. A total of 140 patients with PAAD were eventually enrolled in our study from TCGA database in the end. The excluded and enrolled patients were listed in Table S1. RNA-seq transcriptome data of 167 normal human pancreatic tissues was download from the Genotype-Tissue Expression (GTEx) project (https://xenabrowser.net/). As to GTEx, FPKM values were extracted, and log2(x + 0.001) transformed into FPKM values, then the FPKM values from TCGA and GTEx database were transformed into transcripts per kilobase million (TPM) values. Data of gene expression quantification was integrated from the TCGA database and GTEx project; 140 patients with PAAD and 167 healthy populations were incorporated into our study in the end.

We obtained the list of immune-related genes from import database (https://immport.niaid.nih.gov/), a long-term, sustainable data warehouse developed to promote re-use of immunological data generated by NIAID DAIT and DMID funded investigators. Besides, the copy number of the genes in 140 PAAD patients was download in the UCSC database (https://xenabrowser.net/). Expression of prognosis m6A modulators mRNA levels was analyzed to compare normal and tumor tissues in various cancer types using the Oncomine database (https://www.oncomine.org). The GSE62452 dataset from the GEO database (https://www.ncbi.nlm.nih.gov/geo/) was used for validation.

### Extraction of m6A RNA methylation modulator expression data

We selected m6A RNA methylation modulators which were widely reported in a variety of cancers. Twenty-eight m6A RNA methylation modulators including 10 writers, 16 readers, and 2 erasers were included in this study (Table 1). mRNA expression data of 28 m6A RNA methylation modulators from TCGA and GTEx projects were extracted for further bioinformatics analysis.

**Table 1:** the lists of the 28 m6A RNA methylation regulative factors

Regulators	Full name	Type
METTL3	Methyltransferase like 3	“writers”
METTL14	Methyltransferase like 14	“writers”
METTL16	Methyltransferase like 16	“writers”
WTAP	WT1 associated protein	“writers”
RBM15	RNA binding motif protein 15	“writers”
RBM15B	RNA binding motif protein 15B	“writers”
ZC3H13	Zinc finger CCCH-type containing 13	“writers”
VIRMA	vir like m6A methyltransferase associated	“writers”
CBLL1	Cbl proto-oncogene like 1	“writers”
ZCCHC4	zinc finger CCHC-type containing 4	“writers”
LRPPRC	leucine rich pentatricopeptide repeat containing	“readers”
ELAVL1	ELAV like RNA binding protein 1	“readers”
YTHDC1	YTH domain containing 1	“readers”
YTHDC2	YTH domain containing 2	“readers”
YTHDF1	YTH N6-methyladenosine RNA binding protein 1	“readers”
YTHDF2	YTH N6-methyladenosine RNA binding protein 2	“readers”
YTHDF3	YTH N6-methyladenosine RNA binding protein 3	“readers”
HNRNPC	Heterogeneous nuclear ribonucleoprotein C	“readers”
HNRNPA2B1	Heterogeneous nuclear ribonucleoprotein A2B1	“readers”
EIF3A	Eukaryotic translation initiation factor 3 subunit A	“readers”
EIF3H	Eukaryotic translation initiation factor 3 subunit H	“readers”
IGF2BP1	IGF2 mRNA-binding protein 1	“readers”
IGF2BP2	IGF2 mRNA-binding protein 2	“readers”
IGF2BP3	IGF2 mRNA-binding protein 3	“readers”
CBLL1	Casitas B-lineage lymphoma-transforming sequence-like protein 1	“readers”
PRRC2A	The proline-rich coiled-coil 2A	“readers”
FTO	Fat mass and obesity-associated protein	“erasers”
ALKBH5	α-ketoglutarate-dependent dioxygenase AlkB homolog 5	“erasers”

### Constructing of m6A RNA methylation modulators signature in pancreatic cancer

Twenty-eight m6A RNA methylation modulators were incorporated into univariate Cox proportional hazard regression, then modulators with a *p*-value < 0.1 were involved in the subsequent LASSO regression for further screening, and the selected modulators were applied to conducted prognosis model. Here m6Ascore = Σni = ^1^Coef × xi where Coef represents the coefficient and xi is the expression value of each selected modulator, and nomograph was portrayed to predict the risk of death and the survival rate.

### Pathways and function enrichment analysis

PAAD samples were categorised into high-risk and low-risk groups according to the median of m6Ascore, “limma” package of R was used to analyse the differentially expressed genes (DEGs) with stated threshold values where false discovery rate (FDR) < 0.05 and |Log2 fold change (FC)| > =1.0, all the DEGs was performed in volcan plot by “ggplot2” package in R, intersection of DEGs and immune-related genes from import database was displayed in venn plot by “VennDiagram” package of R software and exhibited in heatmap partly. Then the function of differentially expressed immune-related gens between two group was performed by Gene ontology (GO) enrichment and Kyoto Encyclopedia of Genes and Genomes (KEGG) pathway analyses, visualised by “ggplot2” package in R. KEGG pathways of m6A RNA methylation modulator genes were identified by GESA-3.0.jar software. Gene sets (c2.cp.kegg. v7.4 symbols.gmt) with nominal *p*-value < 0.05 and false discovery rate (FDR) q-value < 0.25 were considered significantly enriched.

### Estimation of tumor-infiltrating immune cell

Estimation proportions of the 22 tumor-infiltrating immune cells (B cells naive, B cells memory, Plasma cells, T cells CD8, T cells CD4 naive, T cells CD4 memory resting, T cells CD4 memory activated, T cells follicular helper, T cells regulatory (Tregs), T cells gamma delta, NK cells resting, NK cells activated, Monocytes, Macrophages M0, Macrophages M1, Macrophages M2, Dendritic cells resting, Dendritic cells activated, Mast cells resting, Mast cells activated, Eosinophils, and Neutrophils) from each sample were calculated by using the “CIBERSORT” (R package) according to the gene expression profiles. The tumor cellularity was inferred through the ESTIMATE algorithm using the “estimate” package in R with default parameters. We acquired ImmuneScore, StromalScore, and ESTIMATEScore of each sample in pancreatic cancer to predict the level of infiltrating stromal and immune cells based on specific gene expression signatures of stromal and immune cells.

### Immune response analysis

The immunophenoscore (IPS) of 140 PAAD patients was obtained from The Cancer Immunome Database (TCIA) (https://www.tcia.at/home), which provides results of comprehensive immunogenomic analyses of next-generation sequencing data (NGS) data for 20 solid cancers from The Cancer Genome Atlas (TCGA) and other data sources. Furthermore, three immunotherapeutic cohorts, including IMvigor210 cohort, TCGA-SKCM cohort, and GSE78220 cohort downloaded from GEO were used to predict the m6Ascore for curative effect of immunotherapy, expression data and detailed clinical information of IMvigor210 cohort were obtained from http://research-pub.gene.com/imvigor210corebiologies, and normalized by the DEseq2R package in R and then the count value was transformed into the TPM value. As to GSE78220 and TCGA-SKCM cohort, the FPKM data of gene expression profiles were also converted to the more comparable TPM value.

### Clinical tissue specimens

A total of six paired pancreatic cancer and adjacent non-tumorous tissue used for immunohistochemistry (IHC) staining were collected from the First Affiliated Hospital of Anhui Medical University. All patients have already provided written informed consent. This work was approved by the Academic Committee of The First Affiliated Hospital of Anhui Medical University and was conducted following the Declaration of Helsinki.

### Immunohistochemistry staining analysis

Collected tissue specimens were formalin-fixed and embedded with paraffin. Tissue sections (4 μm thickness) were used in IHC staining analyses, and the slices were treated with methanol containing 3% hydrogen peroxide to inactivate the endogenous peroxidase and treated with citric acid buffer (pH = 6.0) to obtain optimal antigen recovery. The slices were incubated in 1% bovine serum albumin in phosphate buffer for 30 min to block non-specific binding. In addition, the slices were stained with primary antibody overnight at 4 °C. Then, these sections were subjected to three 5-min mild washing in phosphate buffer saline, followed by staining with secondary antibody (HRP polymer) at 1:200 for 50 min. Diaminobenzidine was applied to the slices before being counterstained with hematoxylin. Finally, the samples were sealed, observed, and photographed by a light microscope.

### Statistical analysis

R 4.0.3 (https://www.r-project.org/) was used for our statistical analyses. Wilcoxon test was used to perform a difference comparison between two group, including differential expression of m6A RNA methylation modulators between cancerous and healthy tissues and the DEGs between high and low m6Ascore group. The relationship between m6Ascore and age, gender, pathological stage and histologic grade were analysed by Chi-square test. Association between m6Ascore and tumor-infiltrating immune cells was computed by Spearman’s correlation. Multiple regression analysis was used to analyse the association between m6A RNA methylation modulators and clinical features. Cox regression analysis was used to examine the prognosis of pancreatic cancer. Kaplan-Meier method was used to analyses the difference in overall survival (OS) between the high-risk and low-risk group. *P*-values less than 0.05 on both sides were statistically significant. Graphics were visualized by R and Graphad prism 8.

## Results

### Expression of m6A RNA methylation modulators was upregulated in patients with PAAD and associated with cancer progression

**Fig. 1:**
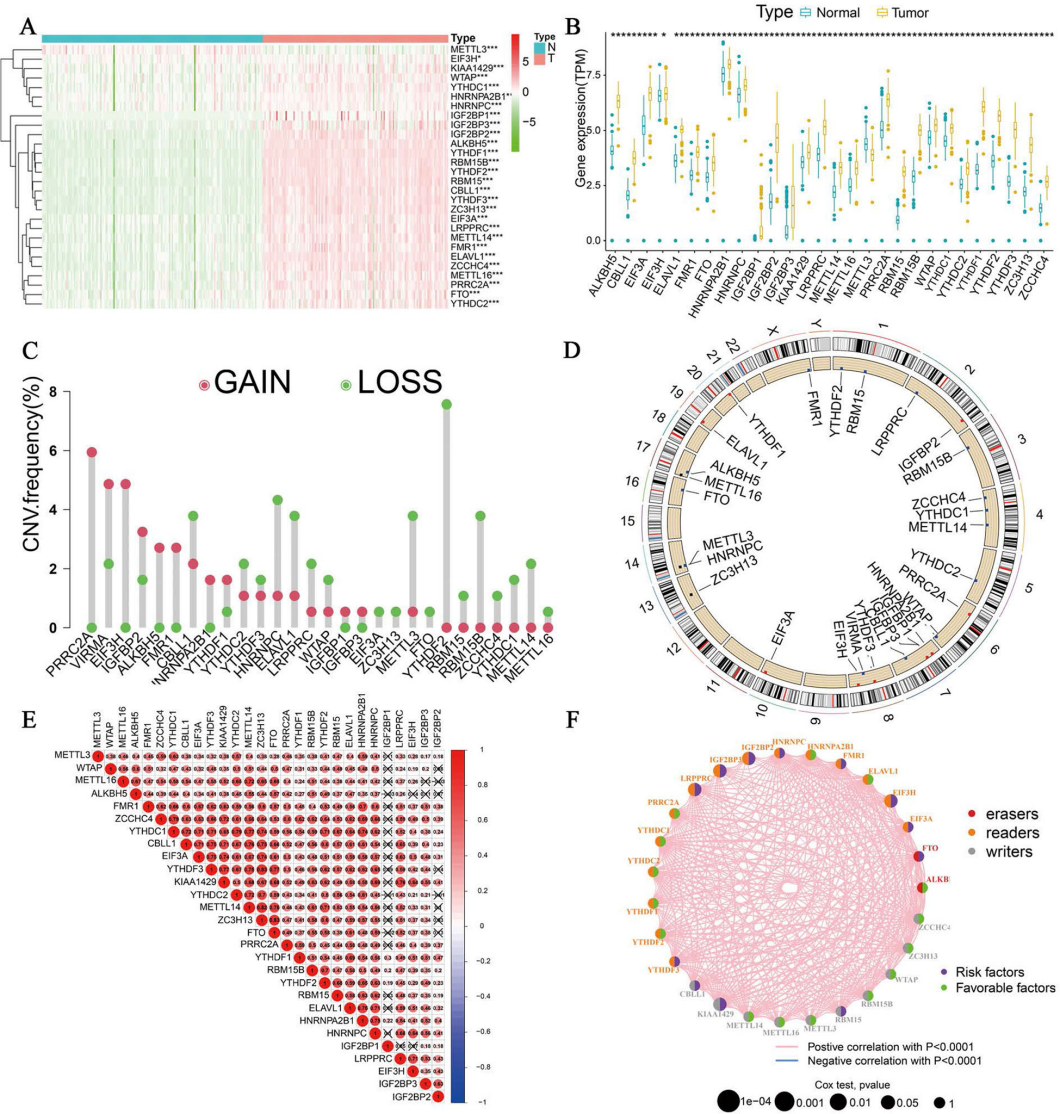
Expression of m6A RNA methylation modulators in 140 patients with PAAD and 167 normal samples based on TCGA and GTEx database. The expression levels of 28 m6A RNA methylation modulators in patients with PAAD presented in **A** a heatmap (green means high expression while blue means low expression and blue means normal samples while pink means tumour samples) and **B** a boxplot (blue means normal samples while red means tumour samples). The association between the expression of the modulators and survival time with statistically significant **C** The CNV variation frequency of 28 m6A regulators in 140 patients with PAAD. **D** The location of CNV alteration of m6A regulators on 23 chromosomes. **E** Spearman’s correlation analysis of the 28 m6A RNA methylation modulators in PAAD patients. **F** Interaction associations among 28 m6A RNA methylation modulators are visualized in the network and the relationship between m6A regulators and prognosis. *p < 0.05, **p < 0.01, ***p < 0.001

Twenty-eight m6A RNA methylation modulators (10 writers, 2 erases and 16 readers) were identified in our study. Expression of 28 m6A RNA methylation modulators was extracted from 167 healthy samples from GTEx database and 140 patients with PAAD integrated from TCGA database (Fig. 1A), All the m6A RNA methylation modulators showed a high expression in pancreatic carcinoma than normal pancreatic tissue except METTL3. The landscape expression of all the 28 m6A RNA methylation modulators is displayed in Fig. 1B. We further analysed the change in copy number of these modulators (Fig. 1C and D), and the alteration frequency of copy number was observed to be common in these modulators, copy number of PRRC2A, KIAA1429, EIF3H, and IGF2BP2 showed a high increment frequency, in contrast, ELAVL1, YTHDF2, and METTL3 displayed a loss of copy number, indicating an abnormal expression of m6A RNA methylation modulators. A positive correlation among m6A RNA methylation modulators was shown in Fig. 1E and F, revealing m6A RNA methylation modulators interacted with each other to influence pancreatic cancer progression. The network results displayed 12 modulators function as risk factors in PAAD patients, including LRPPRC, IGF2BP2, IGF2BP3, HNRNPC, FMR1, EIF3H, EIF3A, FTO, CBLL1, RBM15, YTHDF3, and KIAA1429.

### m6A RNA methylation modulators were associated with clinical characteristic of PAAD

**Fig. 2:**
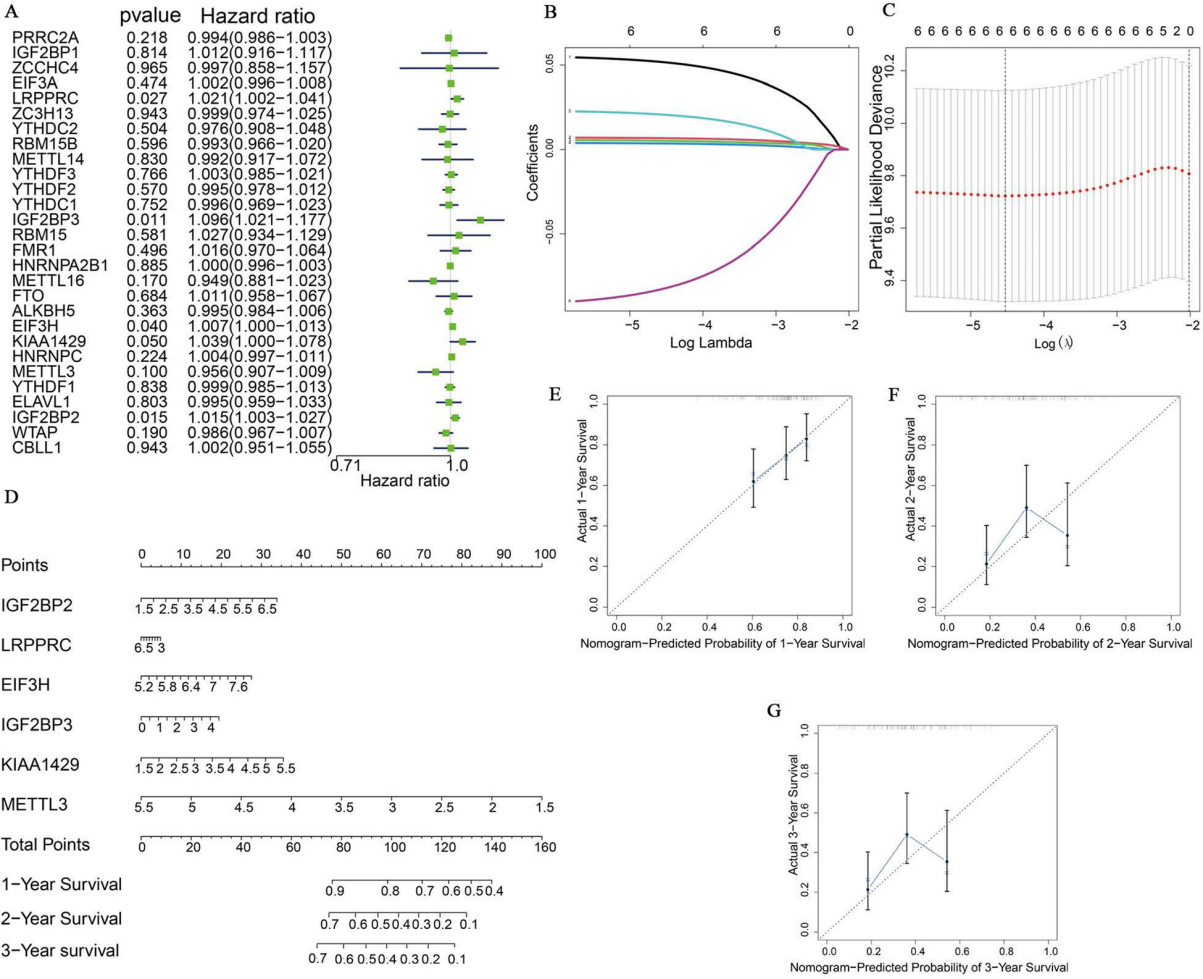
Prognostic value of risk score from m6A RNA methylation modulators expression in PAAD patients. **A** Cox univariate analysis of m6A RNA methylation modulators. **B** and **C** Selection of genes by LASSO Cox regression. **D** Nomogram predicting 1-, 2-, and 3-year survival of patients with PAAD based on the expression of selected m6A RNA methylation modulators. The top row shows the point value for each variable, rows 2–6 indicate the expression of selected m6A RNA methylation modulators, the sum of these values is located on the Total Points axis, and the line drawn downward to the survival axes is used to determine the likelihood of 1-, 2- or 3-year survival. Calibration of nomograms predicting. Calibration of nomograms predicting **E** 1-year, **F** 2-year, and **G** 3-year survival

We built a prognosis prediction model to discover the influence of the 28 m6A RNA methylation modulators in PAAD. First, we conducted a Cox univariate analysis, which showed that the expression of KIAA1429, IGF2BP2, IGF2BP3, METTL3, EIF3H and LRPPRC was correlated with the survival (Fig. 2A). Modulators less than 0.1 in cox univariate analysis, including IGF2BP2, IGF2BP3, LRPPRC, KIAA1429, EIF3H, and METTL3, were subsequently filtrated. Variable selection was performed by LASSO Cox regression and the parameter λ indicated that the most suitable model to predict survival included KIAA1429, IGF2BP2, IGF2BP3, METTL3, EIF3H and LRPPRC with coefficients of − 0.052, 0.007, 0.005, 0.02, 0.004, and − 0.08, respectively (Fig. 2B-C). The expressions of the selected modulators were incorporated to form a nomogram to estimate 1-, 2-, and 3-year OS of pancreatic cancer (Fig. 2D). We used calibration curves to examine the precision of the nomogram (Fig. 2E-G) and observed that all three calibration curves were close to the ideal curve, which indicated an excellent prediction value. To analyze the association between expression of the six selected modulators and clinical parameters, we combined the expression data and clinical parameters of 140 PAAD patients, and the baseline characteristics of these patients are listed in Table 2. The survival curve of the six selected modulators showed high expression of LRPPRC, IGF2BP2, IGF2BP3, EIF3H, and KIAA1429 was related to decreased survival while overexpression of METTL3 displayed a favourable prognosis (Figure S1). We analyzed the relationship between the expression of the six modulators and the clinical characteristic of PAAD, and found a high expression of EIF3H, IG2BP3, IG2BP2, and LRPPRC was related to the maximum tumor dimension, while reduced expression of METTL3 may be associated with the maximum tumor dimension, overexpression of IGF2BP2 and LRPPRC was relevant to a higher histologic grade (Table 3).

**Table 2:** Demographic and clinicopathological parameters of PAAD patients based on TCGA datasets

Clinical characteristics	All	High m6Ascore	low m6Ascore	*P* value
Age	64.54 ± 10.81	63.74 ± 11.68	65.33 ± 9.88	0.387
Gender				0.735
Female	66	34	32	
Male	74	36	38	
Race				0.689
Asian	8	5	3	
White	123	60	63	
Black or African American	5	2	3	
Fustat				< 0.001
Living	63	21	42	
Deceased	77	49	28	
Pathologic stage				0.927
I (IA + IB)	12	6	6	
II (IIA + IIB)	121	60	61	
III+ IV	7	4	3	
T				0.813
T1+ T2	21	10	11	
T3 + T4	119	60	59	
M				0.361
M0	66	34	32	
M1	4	3	1	
N				0.808
N0	37	19	18	
N1(N1a + N1b)	102	50	52	
Histologic grade				0.140
G1-G2	98	45	53	
G3-G4	42	25	17	
Site				
Head of pancreas	110	54	56	0.139
Body of pancreas	11	8	3	
Tail of pancreas	10	6	4	
others	9	2	7	
Maximum tumor dimension	3.75 ± 1.44	4.05 ± 1.72	3.42 ± 0.98	0.013
History of targeted molecular therapy				0.185
YES	75	37	38	
NO	35	22	13	
Alcohol drinking history				0.910
YES	80	40	40	
NO	49	24	25	
History of chronic pancreatitis				0.726
YES	12	7	5	
NO	100	53	47	
History of diabetes				0.157
YES	32	13	19	
NO	85	47	38	
Family history of cancer				0.910
YES	50	21	29	
NO	33	23	10	

Annotation: Numbers that do not add up to 100% are attributable to missing data

**Table 3:** Association between the expression of eight m6A RNA methylation modulators and clinical characteristic in patients with PAAD based on the TCGA datasets

	EIF3H		IGF2BP3		IGF2BP2		KIAA1429		METTL3		LRPPRC	
	down expression	overexpression	down expression	overexpression	down expression	overexpression	down expression	overexpression	down expression	overexpression	down expression	overexpression
N	70	70	70	70	70	70	70	70	70	70	70	70
Age	64.54 ± 10.77	64.53 ± 10.92	66.57 ± 9.71	62.50 ± 11.51	67.46 ± 9.92	61.61 ± 10.93	65.51 ± 10.68	63.56 ± 10.92	65.77 ± 10.62	63.30 ± 10.93	64.66 ± 10.42	64.41 ± 11.26
*P*-value	0.994		0.025		0.001		0.286		0.177		0.895	
Gender												
female	33 (47.14%)	33 (47.14%)	35 (50.00%)	31 (44.29%)	39 (55.71%)	27 (38.57%)	27 (38.57%)	39 (55.71%)	35 (50.00%)	31 (44.29%)	31 (44.29%)	35 (50.00%)
male	37 (52.86%)	37 (52.86%)	35 (50.00%)	39 (55.71%)	31 (44.29%)	43 (61.43%)	43 (61.43%)	31 (44.29%)	35 (50.00%)	39 (55.71%)	39 (55.71%)	35 (50.00%)
*P*-value	1.000		0.498		0.042		0.042		0.498		0.498	
Maximum tumor dimension	3.44 ± 1.03	4.04 ± 1.70	3.40 ± 0.96	4.10 ± 1.74	3.37 ± 0.95	4.15 ± 1.74	3.79 ± 1.57	3.71 ± 1.30	3.52 ± 1.14	3.98 ± 1.68	3.49 ± 1.07	3.99 ± 1.69
*P*-value	0.016		0.006		0.002		0.755		0.069		0.050	
Stage												
StagI (IA + IB)	6 (8.57%)	6 (8.57%)	8 (11.43%)	4 (5.71%)	8 (11.43%)	4 (5.71%)	4 (5.71%)	8 (11.43%)	7 (10.00%)	5 (7.14%)	6 (8.57%)	6 (8.57%)
StagII (IIA + IIB)	62 (88.57%)	59 (84.29%)	59 (84.29%)	62 (88.57%)	59 (84.29%)	62 (88.57%)	62 (88.57%)	59 (84.29%)	58 (82.86%)	63 (90.00%)	60 (85.71%)	61 (87.14%)
StagIII+IV	2 (2.86%)	5 (7.14%)	3 (4.29%)	4 (5.71%)	3 (4.29%)	4 (5.71%)	4 (5.71%)	3 (4.29%)	5 (7.14%)	2 (2.86%)	4 (5.71%)	3 (4.29%)
*P*-value	0.507		0.461		0.461		0.461		0.401		0.927	
Grade												
G1 + G2	51 (72.86%)	47 (67.14%)	51 (72.86%)	47 (67.14%)	59 (84.29%)	39 (55.71%)	52 (74.29%)	46 (65.71%)	52 (74.29%)	46 (65.71%)	55 (78.57%)	43 (61.43%)
G3 + G4	19 (27.14%)	23 (32.86%)	19 (27.14%)	23 (32.86%)	11 (15.71%)	31 (44.29%)	18 (25.71%)	24 (34.29%)	18 (25.71%)	24 (34.29%)	15 (21.43%)	27 (38.57%)
*P*-value	0.461		0.461		< 0.001		0.268		0.268		0.027	

### m6Ascore correlated with clinical prognostic of patient with PAAD and was an independent prognostic indicator

**Fig. 3:**
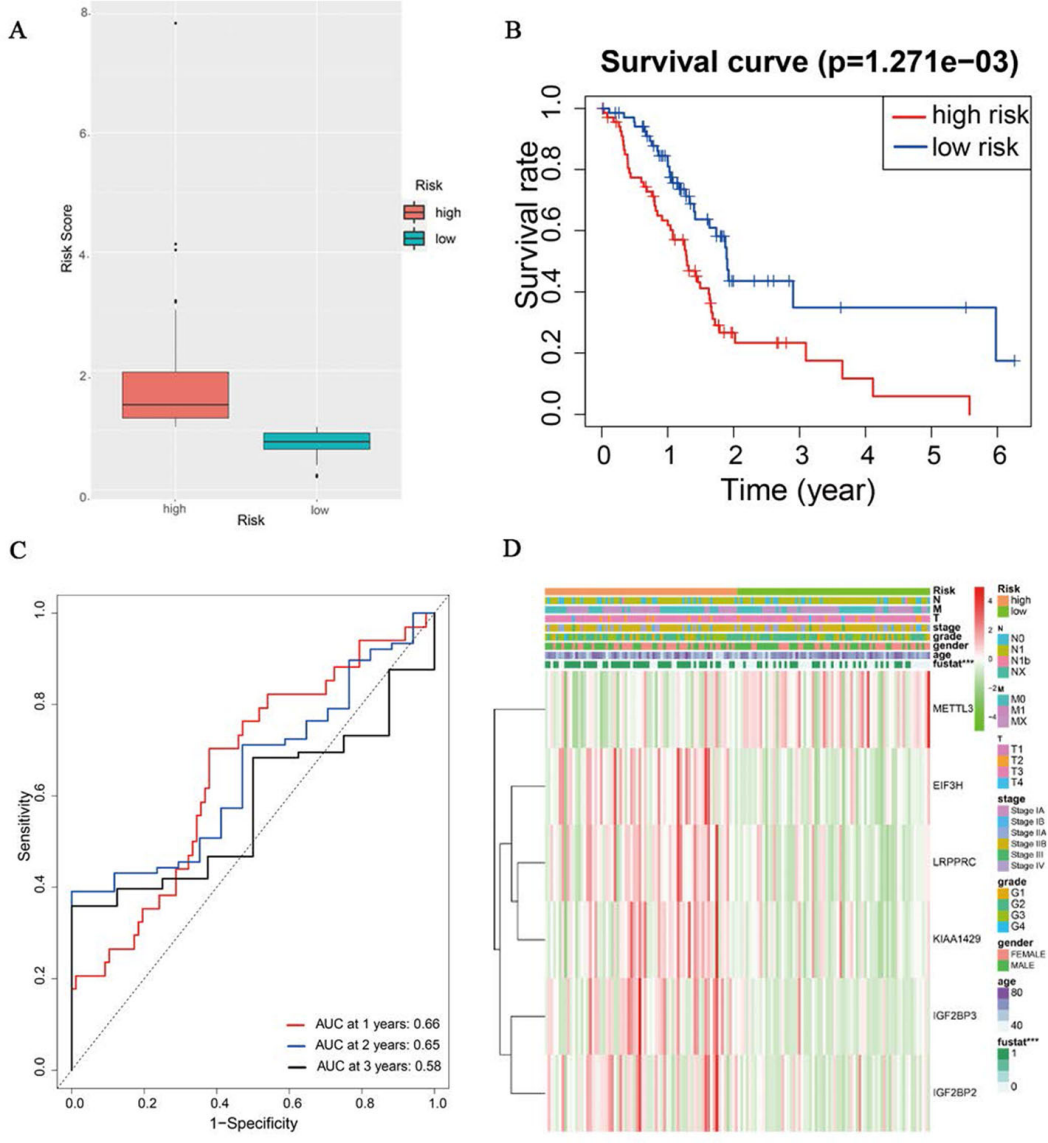
Relationship between m6A risk score and OS of PAAD patients. **A** landscape of risk grouped by risk score. **B** Survival analyses for low and high m6Ascore patient groups using Kaplan-Meier curves. **C** Time-dependent ROC analysis of risk score in predicting prognoses. **D** The difference of clinical features between high and low risk group

The expression of the six m6A RNA methylation modulators was merged using the m6Ascore formula and defined as a risk score, patients were then divided into high- and low-risk groups (Fig. 3A). The result of survival curve predicted that PAAD patients with high m6Ascore had a 1,2 and 3-year OS rate of 61.7, 23.3 and 17.5%, respectively, in patients with low-risk, 1,2 and 3-year OS rate was 82.7, 44.0 and 32% respectively. The risk score model showed a good prediction of 1,2 and 3-year OS, survival curve showed worse OS in high-risk patients than patients in the low-risk group (Fig. 3B and C). Then we investigated the relationship between the risk score and the clinical characteristic of patients with PAAD. Risk score was associated with living status and maximum tumor dimension of patients with PAAD (Fig. 3D, Table 2).

**Fig. 4:**
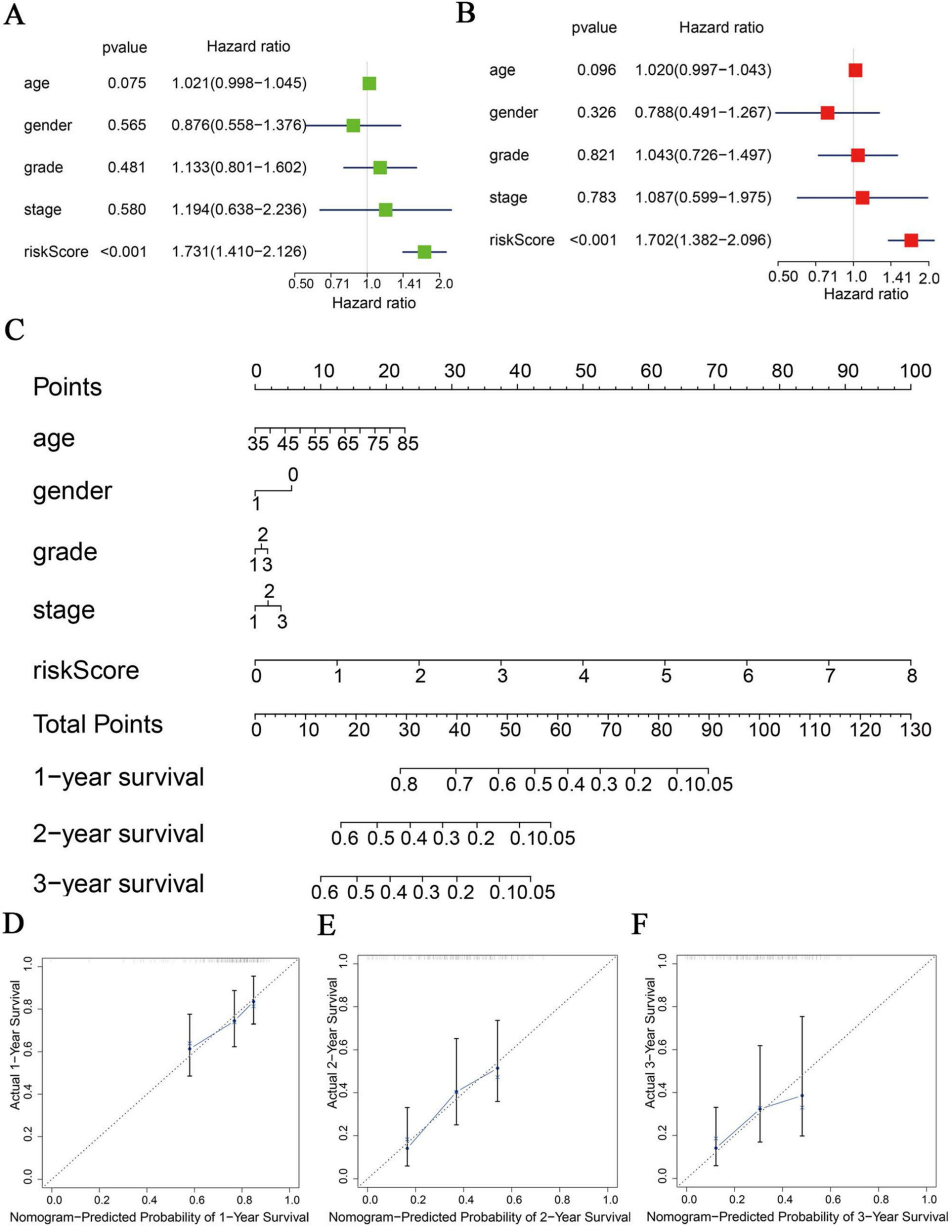
The risk score is an independent prognostic indicator of PAAD patients. **A** Univariate and **B** multivariate Cox regression analysis of the association between clinical and pathological factors, including the risk score and OS of PAAD patients. **C** Nomogram based on age, gender, tumour grade and stage, and risk score. Calibration of nomograms predicting **D** 1-year, **E** 2-year, and **F** 3-year survival

We subsequently hypothesised that risk score could be an independent prognostic indicator and then incorporated risk score and relevant clinical and pathological factors including age, gender, tumour grade and stage into univariate and multivariate Cox regression to test our hypothesis. Univariate Cox regression revealed that risk score was significantly linked with OS (Fig. 4A). Moreover, risk score was still associated with OS in a multivariate Cox regression with HR = 1.702(95%CI:1.382–2.096, *P* < 0.001) (Fig. 4B). These results indicated that m6Ascore could be an independent prognostic indicator of PAAD, the death rate of patients with high-risk score was nearly two times higher than those in the low-risk group, we formed a nomogram with age, gender, tumour grade, stage, and risk core to predict 1-, 2-, and 3-year survival of patients with PAAD (Fig. 4C), calibration curves showed a good prediction value of our risk model (Fig. 4D-F).

### The function analysis and the pathways of the genes that enriched in the group of PAAD patients with high-risk and low-risk

**Fig. 5:**
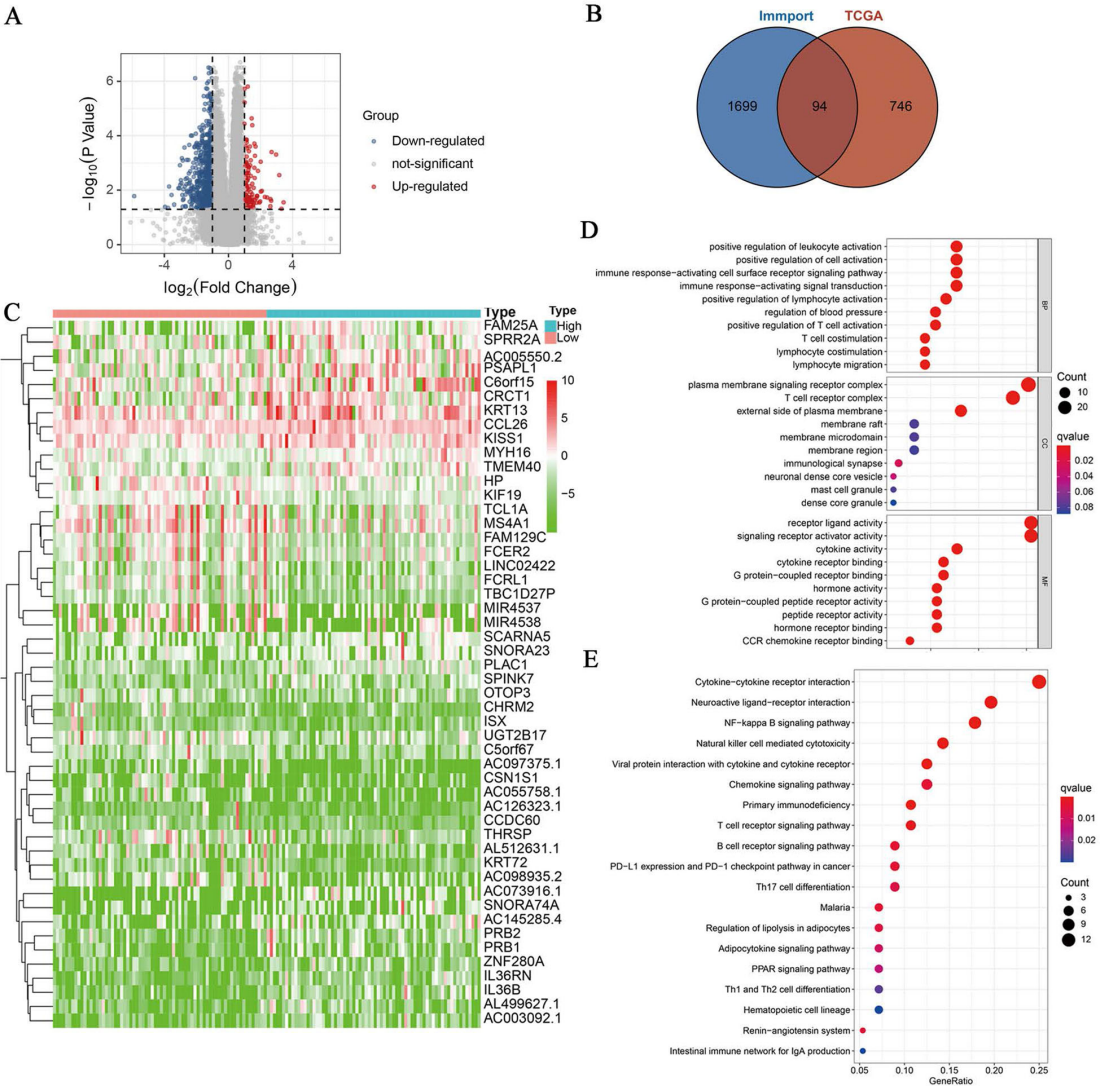
Identifying differentially expressed immune-related genes and enrichment analysis. **A** Differentially expressed genes between high and low m6Ascore group and visualized in volcano plot. **B** Intersection between differentially expressed genes from TCGA database and immune-related genes from Immport database showed in venn diagram. **C** Heatmap of the top 50 intersection immune-related genes with higher logFC. **D** Gene ontology enrichment analysis of intersection immune-related genes, BP biology process; CC cellular component; MF molecular function. **E** KEGG pathway enrichment analysis of intersection immune-related genes

Further analysis was conducted to determine the different expressed genes between high and low m6Ascore patients, all the genes between the two groups were presented in volcano plot (Fig. 5A), around 840 genes showed differential expression from TCGA, 1793 immune related-genes were acquired from immport database, the venn plot showed 94 immune related-genes among the different genes, including 6 upregulated and 88 downregulated genes in patients with high m6Ascore (Fig. 5B). the top 50 different immune related-genes were displayed in heatmap according to the following filter: false discovery rate (FDR) < 0.05 and |Log2 fold change (FC)| > =1.0 (Fig. 5C), IL36B, IL36RN and CCL26 were the top 3 downregulated gene in low m6Ascore group with more remarkable Log2 fold change, whereas CD19, AGTR2 and TNFRSF13C were the top 3 overexpression in patients with high m6Ascore (Fig. 5C). The GO functional enrichment analysis was carried out to exhibit the function of 94 immune related-genes between high m6Ascore and low m6Ascore group, a total of 344 GO terms were enriched, including 295 biological process terms, 5 cellular component terms and 44 molecular function terms. Kegg pathway analysis was performed to present the related biological pathways among these DEGs, a total of 19 Kegg pathways were enriched among these DEGs, the top 10 GO terms of three functional groups and all enriched Kegg pathways were listed in our results with the most significant *P* values and bigger counts (Fig. 5D-E). DEGs take part in the modulation of T cell costimulation, activating cell surface receptor signaling, activating signal transduction, and join the plasma membrane signaling receptor complex, T cell receptor complex and external side of the plasma membrane influenced receptor-ligand, signaling receptor activator, and cytokine activity, on the other hand. Analysis of Kegg showed an enrichment in cytokine-cytokine receptor interaction, NF-kappa B signaling pathway, PD-L1 expression and PD-1 checkpoint pathway in cancer progression.

**Fig. 6:**
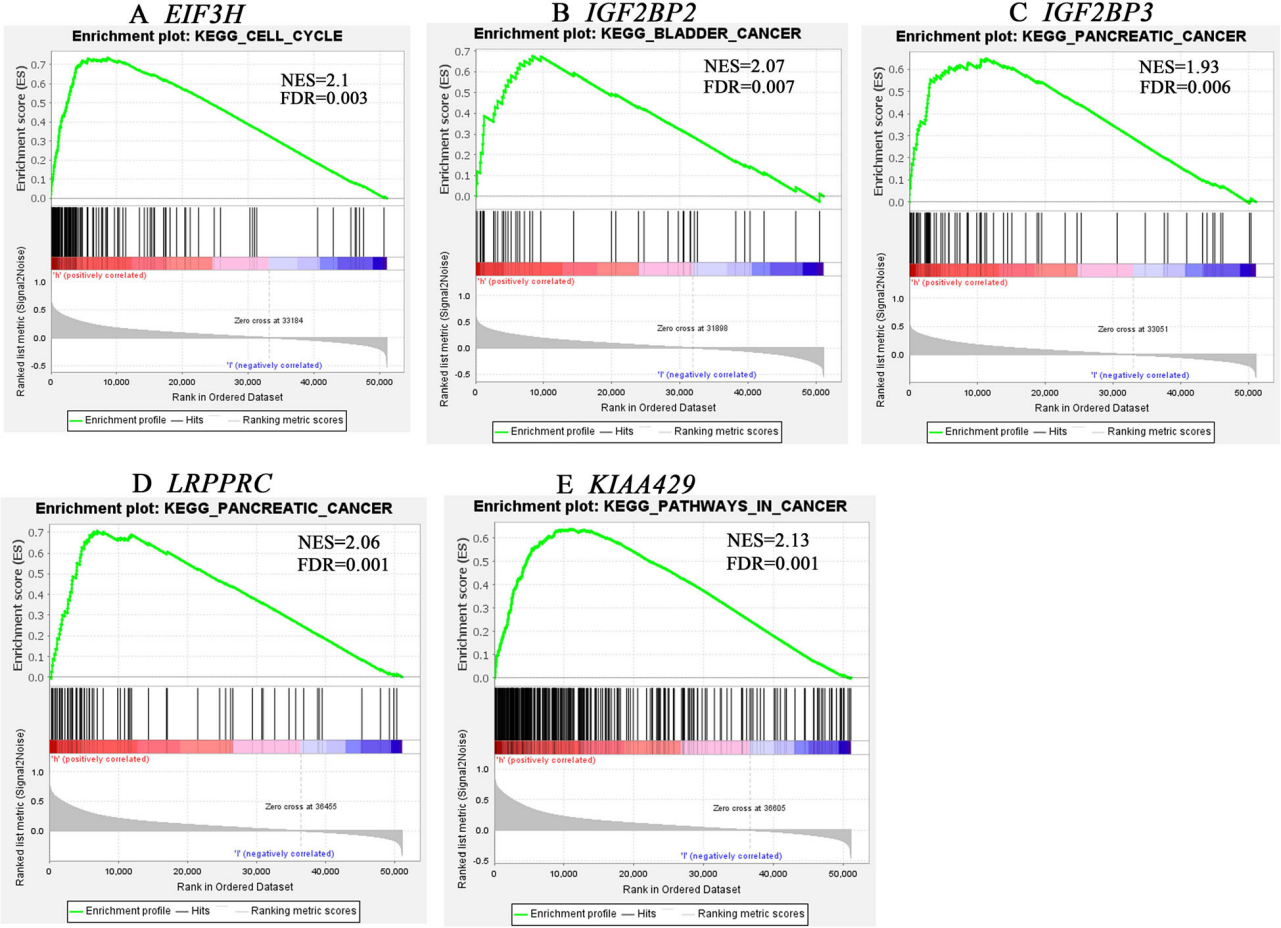
The associated KEGG pathways of m6A RNA methylation genes by GSEA. **A** *EIF3H*. **B** *IGF2BP2*. **C** *IGF2BP3*. **D** *LRPPRC*. **E** *KIAA1429*

To identify the potential biological function of the m6A RNA methylation modulators that predicted the OS of patients with PAAD, we used GSEA to search for the associated Kegg pathways of m6A RNA methylation modulator genes (Fig. 6A-G). The enrichment score (ES), FDR, and nominal *p*-value were shown for each gene in Table 4. Although high expression of *EIF3H* expression was linked to cell cycle, no gene sets were significantly enriched in patients with high expression of *METTL13*. Gene sets were significantly enriched in Kegg pancreatic cancer with an upregulation of *LRPPRC* and *IGF2BP3,* we also observed a high expression of *KIAA1429* in the Kegg pathways in cancer.

**Table 4:** gene set enrichment analysis for six m6A modulators with KEGG pathway using GSEA

Gene name	KEGG pathway	ES	NES	NOM p-val	FDR q-val
*EIF3H*	KEGG_CELL_CYCLE	0.73	2.1	0.000	0.003
*IGF2BP2*	KEGG_BLADDER_ CANCER	0.68	2.07	0.000	0.007
*IGF2BP3*	KEGG_PANCREATIC_CANCER	0.65	1.93	0.000	0.006
*LRPPRC*	KEGG_PANCREATIC_CANCER	0.71	2.06	0.000	0.001
*VIRMA*	KEGG_PATHWAYS IN _CANCER	0.64	2.13	0.000	0.001

### m6Ascore was related with the immune cell infiltration of PAAD

**Fig. 7:**
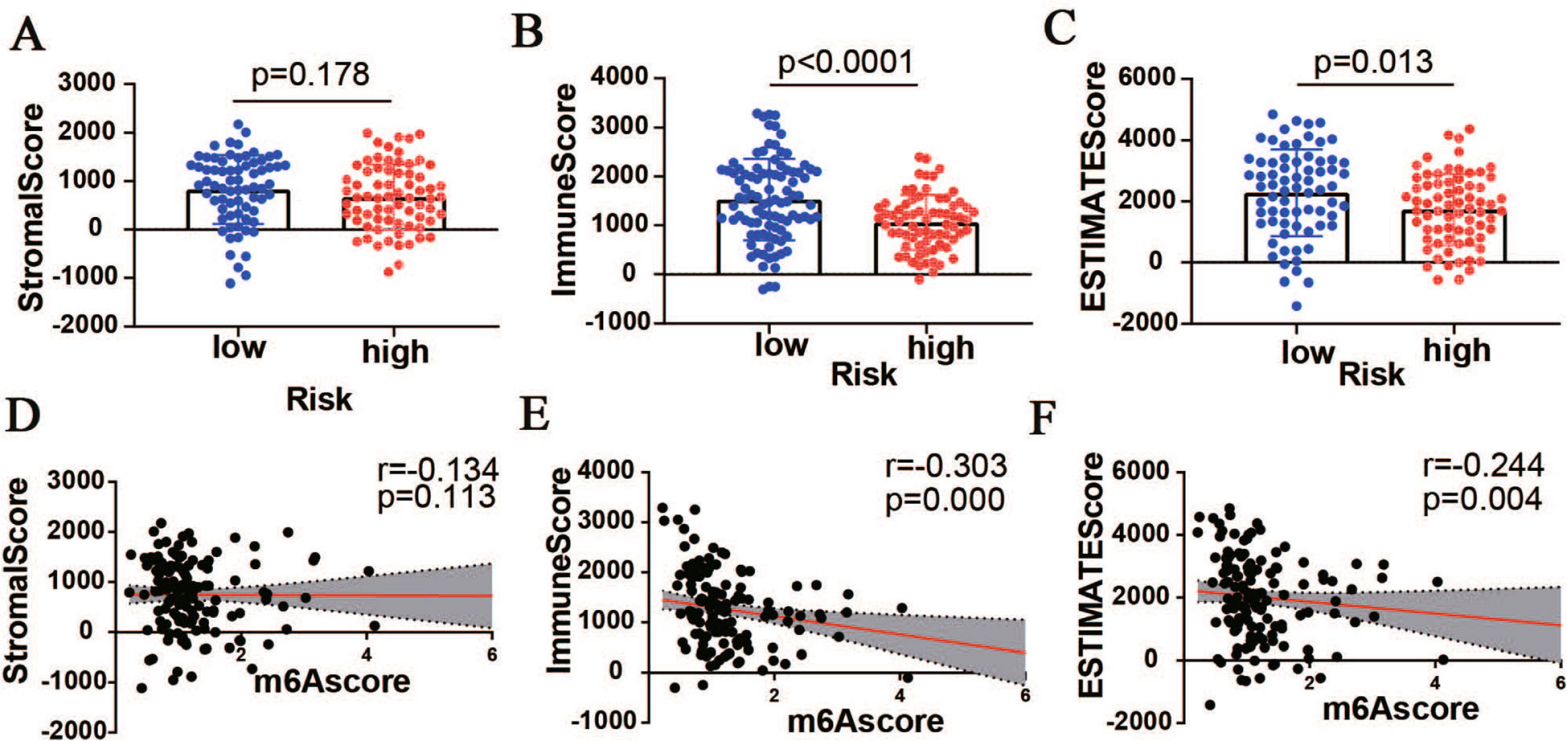
The association between tumor microenvironment and m6Ascore. Discrepancy of stromalscore (**A**), immunescore (**B**) and estimatescore (**C**) in two groups. Spearman’s correlation analysis of m6Ascore with tumor microenvironment. **D** stromalscore. **E** immunescore. **F** estimatedscore

**Fig. 8:**
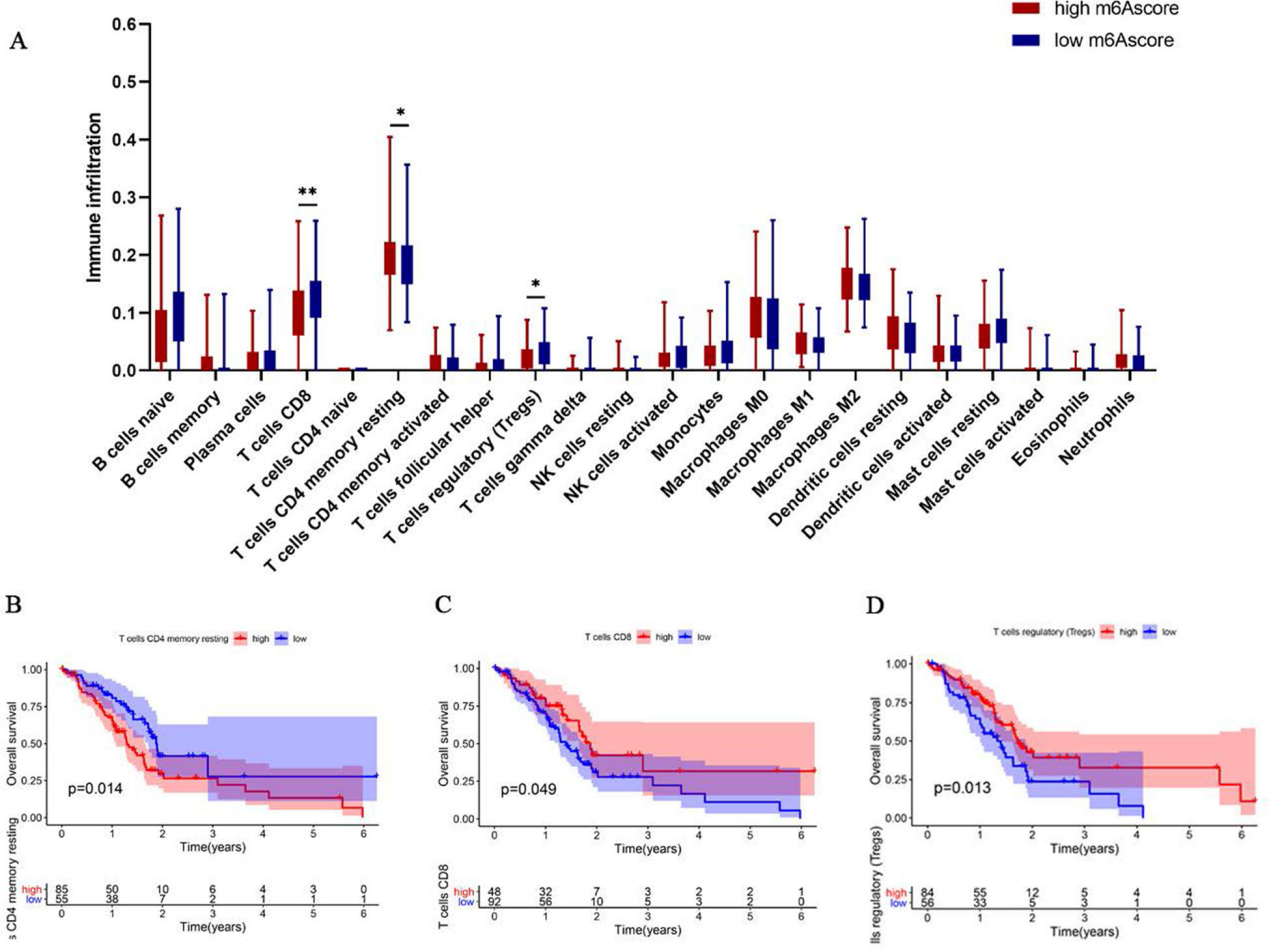
The different tumor-infiltrating immune cells between high and low m6Ascore patients with PAAD. **A** Profiles of 22 type of tumor-infiltrating immune cells in two group. The relationship between OS and **A** resting memory CD4+ T cells, **B** CD8+ T cells, **C** Tregs cells

The composition of infiltrating immune cells between high and low m6Ascore group wsa explored by CIBERSORT algorithm. As for the tumor immune microenvironment in PAAD, immune scores and estimate scores were higher in PAAD patients with low m6Ascore (Fig. 7A-C), furthermore, we found a low reverse relationship between m6Ascore and immune score with *r* = − 0.303 (Fig. 7D-F), which pointed out that tumor purity was higher in the high m6Ascore group, indicating that patients with an unfavourable prognosis in the high m6Ascore group associated with the variation in tumor immune microenvironment of PAAD. Then we provided an insight into the correlations of m6Ascore with the 22 type of immune cells (Fig. 8A), patients with high m6Ascore had lower infiltration of Tregs and CD8^+^T cells but a higher resting CD4^+^ T infiltration, other immune cells showed no difference between the high and low groups. Furthermore, low CD8^+^T cells were significantly related to a poor OS, indicating that exhaustion of CD8^+^T cells that may lead to a poor OS in the high-risk group (Fig. 8B-D).

**Fig. 9:**
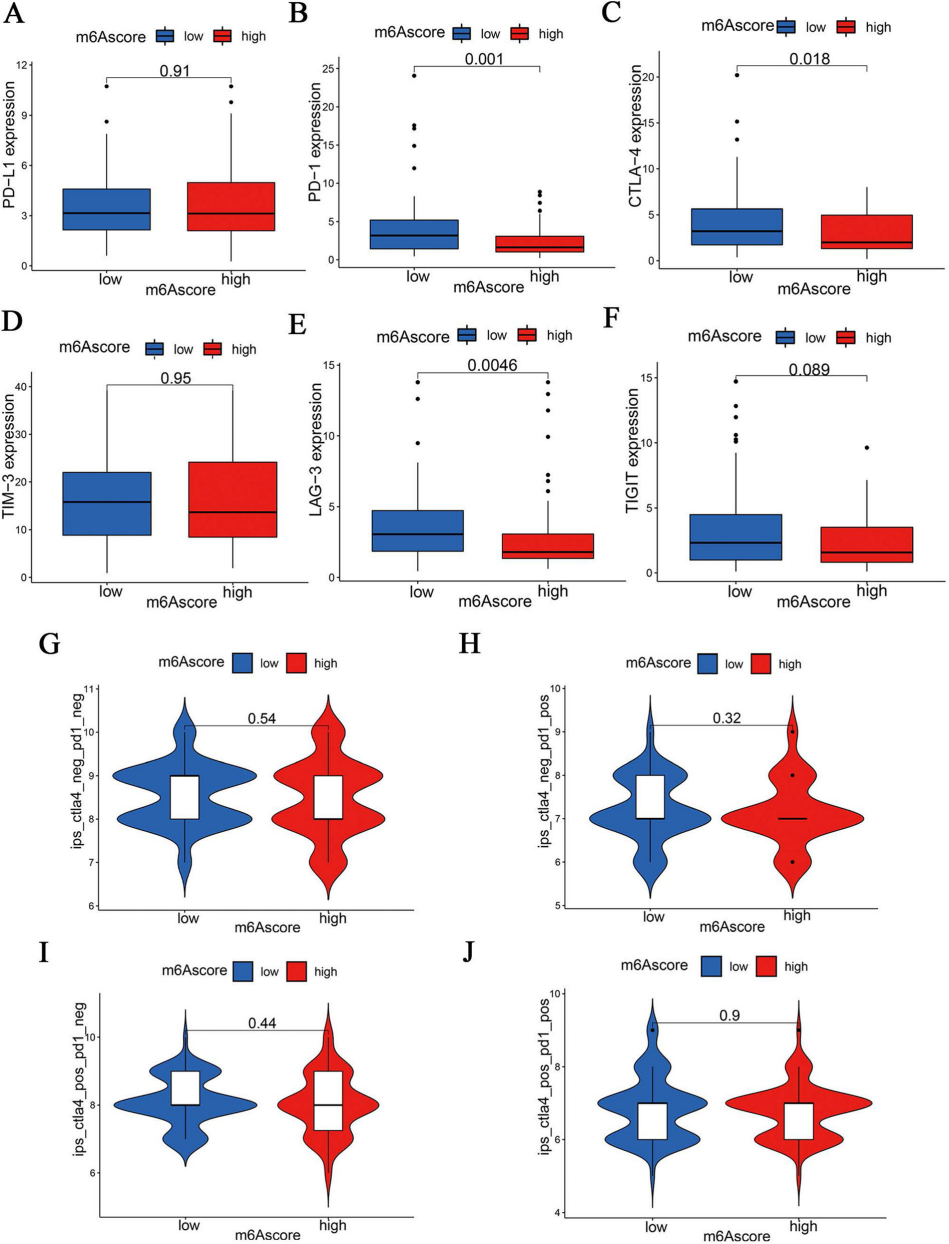
IPS and immunotherapy gene expression analysis. The levels of immune checkpoints molecules including PD- L1 (**A**), PD-1 (**B**), CTLA-4 (**C**), TIM-3 (**D**), LAG-3 (**E**), and TIGIT (**F**) in low-risk and high-risk groups. The association between IPS and the m6Ascore in PAAD patients based on TCIA database, **H** CTLA4^−^ PD1^−^ **I** CTLA4^−^ PD1^+^ **J** CTLA4^+^ PD1^−^ CTLA4^+^PD1^+^

To predict the pancreatic cancer patients’ response to immunotherapy, we further investigated the difference in the expression of immune checkpoints (*PD-1, PD-L1, CTLA-4, LAG3, TIGIT and TIM-3*) between patients with low and high m6Ascore (Fig. 9A-F). Our results showed that patients with a low m6Ascore displayed a high abundance of *PD-1, CTLA-4* and *LAG3*. IPS of 140 PAAD patients got from TCIA database was considered to be a superior predictor of response to anti-cytotoxic T lymphocyte antigen-4 (CTLA-4) and anti-programmed cell death protein 1 (anti-PD-1) antibodies, but IPS showed no discrepancy in two groups (Fig. 9H-K). Furthermore, three immunotherapy cohorts, including the IMvigor210 cohort, GSE78220 cohort and TCGA-SKCM cohort, were also used to investigate whether m6Ascore could predict patients’ responses to anti-PD-L1 therapy. in both GSE78220 and TCGA-SKCM cohort, patients with a low m6Ascore showed a high proportion of response to anti-PD-L1, but survival rate showed no difference in patients with high or low m6Ascore among all three anti-PD-L1 immunotherapy cohorts. Therapeutic effects of immunotherapies in patients with PAAD need to be verified using further studies because of the discrepancy in prediction results (Figure S2).

### External validation of six key prognostic m6A RNA modulators

External pancreatic cancer databases from oncomine and GSE62452 were used to validate the expression of the six m6A RNA modulators in our prognosis model. In the oncomine database, expression of KIAA1429, LRPPRC, IGF2BP2, IGF2BP3, and EIF3H was higher in pancreatic cancer than normal tissues (Figure S3 A-F). In the GSE62452 dataset, we found a high expression of IGF2BP2 and IGF2BP3 in pancreatic tumor tissues compared with adjacent non-tumor tissue (Figure S4 A-F). Then we applied the risk score algorithm into the validation cohort to further verify the predictive value of the risk signature. In GSE62452 dataset, we also found that patients with a high m6Ascore showed poor prognosis, and the result from ROC analysis displayed that risk score had a predictive accuracy of prognosis, the AUC of 1-year, 3-year and 5-year survival was 0.61, 0.71, and 0.74, respectively (Figure S4 G-H).

**Fig. 10:**
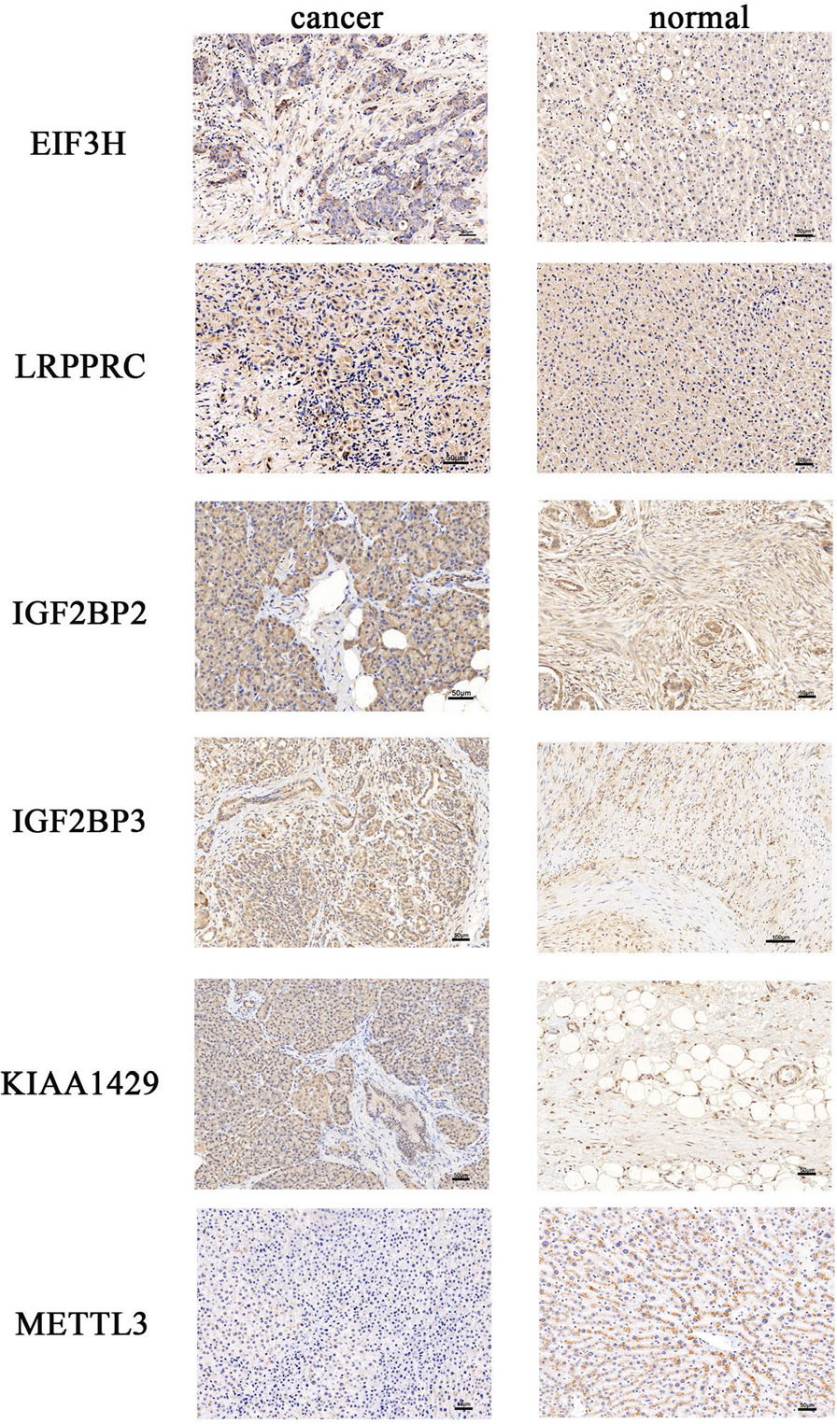
IHC analysis of six m6A modulators in pancreatic cancer tissues and adjacent normal tissues

IHC staining of six paired pancreatic cancer and adjacent non-tumorous tissues was used to validate the protein expression of the six prognosis genes (Fig. 10). The Compared with adjacent non-tumorous tissues, we found a higher level of EIF3H, IGF2BP2, IGF2BP3, KIAA1429 in all six pancreatic tumor tissues, LRPPRC was overexpressed in five pancreatic tumor tissues, while the expression of METTL3 decreased in four pancreatic tumor tissues.

## Discussion

The high death rate of pancreatic cancer has been a continuous challenge in the field, it can progress rapidly with only mild symptoms, and there may be a multitude of causes, including a combination of lifestyle, environment, and genetic mutation. Surgery is the preferred treatment for resectable pancreatic cancer with a poor prognosis. Pancreatic cancer is generally insensitive to both chemotherapy and radiotherapy. Therefore, new treatment strategies focusing on the immunotherapy and targeted therapy are urgently needed for patients with pancreatic cancer. m6A is involved in the development of many cancers and some studies have shown its potential in the treatment of diverse cancers. In this study, we explored the function of multiple m6A methylation modulators in pancreatic cancer, identified the role of m6A methylation modulators signature in the TME cell infiltration and estimated the therapeutic effects of immunotherapy in pancreatic cancer.

We analysed 28 widely reported m6A RNA methylation modulators in pancreatic cancer according to the TCGA and GTEx database, a higher expression of these modulators except METTL3 was found in patients with PAAD compared to the normal tissues, suggesting an alterant level of m6A RNA methylation may play a carcinogenic role in pancreatic cancer. The prognosis model was conducted based on the expression of the selected methylation modulators, including KIAA1429, IGF2BP2, IGF2BP3, LRPPRC, METTL3, and EIF3H in our study. A similar study published in 2020 conducted a prognosis model based on the expression of KIAA1429, IGF2BP2, IGF2BP3, HNPNPC, METTL3, and YTHDF1. The discrepancy could be due to the difference of enrolled samples, but the similar finding was that METTL3 acts as a favourable factor while KIAA1429, IGF2BP2 and IGF2BP3 were risk factors in both pieces of research [36]. An overexpression of METTL3 in the MIA-PaCa-2 and BxPC-3 pancreatic cancer cell lines was involved in pancreatic carcinogenesis and promoted proliferation and invasion of pancreatic cancer cell lines in Xia T’s study [37]. Taketo K et al. pointed out that METTL3-depleted cells showed higher sensitivity to chemo- and radioresistance in pancreatic cancer cells [38], which was adverse with our analysis. IGF2BP2 and IGF2BP3, members of the mammalian IGF2 mRNA-binding protein family, were found correlated with an overall poor prognosis and metastasis of various cancer [39]. We found a higher pathologic stage and histologic grade associated with the overexpression of IGF2BP2 and IGF2BP3 in our study. The results from GSEA showed that overexpression of IGF2BP2 and IGF2BP3 was related to the progression of cancer, IGF2BP3 may involve rearrangement of peripheral actin, which led to the formation of additional membrane protrusions, promoting cell invasiveness and tumor metastasis of pancreatic cancer [40], IGF2BP2 was confirmed to be an oncogenic factor; miR-141 was a downstream regulatory gene in the progression of pancreatic cancer in a previous study [41]. EIF3H showed a function on cell cycle from GSEA in our study; however, the specific mechanism in PAAD need to be confirmed. Further, LRPPRC was a multifunctional protein involved in energy metabolism, and we found high LRPPRC enriched in pancreatic cancer, and it may play an essential role in pancreatic tumorigenesis by connecting an oncogenic gene expression with energy production. KIAA1429 overexpression is seen in many types of cancer that drive tumor growth and liver cancer cells’ metastasis by targeting GATA3 [42].

Tumorigenesis is a process that relies on the coordination of multiple factors, results showed that risk score was an independent prognosis indicator, and the combined expression of the selected modulators had a good prediction of 1-, 2-, and 3-year OS of PAAD, patients with high-risk score related with an unfavourable OS. Previous reports described m6A regulator-mediated methylation modification patterns played a non-negligible role in tumor microenvironment infiltration in gastric cancer [43]. Comparing the immune-related genes between high and low m6Ascore group, we found *IL36B and IL36RN* exhibited a higher level in the high m6Ascore group where *IL36B* was a gene involved TH17 pathway and played a role in polarizing T-helper responses. *IL36RN* encoded an interleukin-36-receptor antagonist [44] in renal cell carcinoma cells, and its overexpression inhibited proliferation, migration, invasion, and colony formation of tumor cells by suppressing β-catenin [45]. *CD19,* a target of chimeric antigen receptor (CAR) T-cell immunotherapy, was downregulated in PAAD patients with low m6Ascore. The results of GO and KEGG pointed out that different immune-related genes participated in the activation and differentiation of T cell and other immune-related pathways, revealing a significant role of m6Ascore signature in tumor’s immune environment. Besides, patients with high m6Ascore had a lower immune score and estimated score than patients with low m6Ascore, while a recent study declared that gliomas patients with high m6Ascore had a more abundance of immune infiltration with higher immune score and stromal score, which was conversed in PAAD [46]. As to the discrepancy of immune cell infiltration, patients with low m6Ascore had an elevation of Tregs and CD8^+^T cells but a low level of resting memory CD4^+^T cells, indicating an association between m6A modification and tumor immunity of PAAD. Moreover, a high level of CD8^+^T cell presented a high survival rate, where exhaustion of CD8^+^T cell may lead to an unfavourable OS in patients with high m6Ascore. Similar results in Han D’s study exhibited a deficiency of YTHDF1 that promoted the antigen-specific activation of CD8+ T cells and attributed to the enhanced capability of DCs to present tumor neoantigens [35]. Increasing evidence had shown that m6A RNA methylation modification plays a role in tumor immunity. Previous research reported that conduction of m6A signature was beneficial to reflect tumor microenvironment features of lung adenocarcinoma in different cohorts [47].

Immunotherapy had been shown been as an effective therapeutic strategy in diverse cancers, however, some patients faced an unsatisfactory effect due to individual variation. TME is composed of tumor vessels and different immune microenvironments, patients have different therapeutic responses due to the discrepancy of their structure. Checkpoint inhibitors have been the most mature and sufficient clinical research and the most widely used of tumor immunotherapy. Immune checkpoint therapy is a therapeutic method to inhibit tumor cells by regulating the activity of T cells through a series of pathways such as co-inhibition or co-stimulation. Various immune checkpoints have been used to treat PAAD, however, PD-1/PD-L1 and CTLA-4 blockade observed limited effect in PAAD treatment [48, 49]. We further predicted the efficacy of PD-1/PD-L1, CTLA-4, LAG-3, TIM-3 and TIGIT, and found a higher expression of PD-1, CTLA-4, and LAG-3 in the low m6Ascore group. Nevertheless, there was no difference in IPS between the two groups, and the estimated results from three immunotherapy cohorts indicated no association between m6Ascore and the curative effect of immunotherapy. Immunotherapy in pancreatic cancer is conflicted, and it needs more clinical trials to confirm the curative effect. For example, a phase I trial included one pancreatic patient who failed to show any efficacy with a treatment of anti-PD-L1 (MPDL-3280A) therapy [50]. In contrast, another clinical trial that enrolled 32 patients with pancreatic cancer showed the best response to MK-3475 and disease-free progression for 20 weeks after anti-PD-1 therapy [51]. Therapy targets immune checkpoints in pancreatic cancer showed a poor effect, and more efforts are required to overcome resistance to PD-1/PD-L1 targeted immunotherapy.

In general, our research found a discrepancy of m6A RNA modification modulators in pancreatic cancer based on TCGA and GTEx databased, and found an association between the expression of m6A RNA modification modulators and clinical characteristic. We conducted an m6A risk model and observed that high m6Ascore presented an unfavourable prognosis in PAAD patients. Besides, patients with high m6Ascore had a lower level of infiltration of CD8+ T cells, suggesting that m6A RNA modification may related to tumor microenvironment. However, the inadequate difference in IPS and m6Ascore showed no function in evaluating the therapeutic effects of checkpoint inhibitors treatment in PAAD. Furthermore, the exact mechanism of m6A modulators needs to be elucidated with future research.

## Conclusion

m6A RNA methylation modulators play an important role in the progression of cancer. In this study, we generated a prediction model using 28 m6A RNA methylation modulators in PAAD and identified their association with the prognosis, and the prediction model was related to immune cell infiltration of PAAD. However, the function of the m6Ascore to evaluate the curative effect of immunotherapy needs to be figured out with further studies. Our study highlighted the involvement of m6A in pancreatic cancer opening up potential new research avenues and it also can be a potential measurement to diagnostic pancreatic cancer and to steer therapeutic decision making.

## Supplementary Information

**Additional file 1: Table S1.** The information of excluded and enrolled samples of PAAD from TCGA database.

**Additional file 2: Figure S1.** The association between the expression of the modulators and survival time with statistically significant (A) METTL3. (B) IGF2BP3. (C) IGF2BP2. (D) KIAA1429. (E) EIF3H. (F) LRPPRC. **Figure S2.** Predictive value of m6Ascore in anti-PD-1/L1 immunotherapy based on three immunotherapeutic cohorts. (A) Survival analyses for patients with high or low m6Ascore in GSE78220 cohort. (B) The proportion of patients with response to anti-PD-1/L1 immunotherapy in patients with high or low m6Ascore in GSE78220 cohort. (C) Survival analyses for patients with high or low m6Ascore in TCGA-SKCM cohort (D) The proportion of patients with response to anti-PD-1/L1 immunotherapy in patients with high or low m6Ascore in TCGA-SKCM cohort. (E) Survival analyses for patients with high or low m6Ascore in IMvigor210 cohort (F) The proportion of patients with response to anti-PD-1/L1 immunotherapy in patients with high or low m6Ascore in IMvigor210 cohort. **Figure S3.** Expression of of six m6A modulators in pancreatic cancer tissues and adjacent normal tissues regarding to oncomine database (A) EIF3H. (B) IGF2BP2. (C) IGF2BP3. (D) KIAA1429. (E) METTL3. (F) LRPPRC. **Figure S4.** External Validation of six Key Prognostic m6A RNA modulators in GSE62452 dataset. Expression of of six m6A modulators in pancreatic cancer tissues and adjacent normal tissues based on GSE62452 dataset. (A) METTL3. (B) LRPPRC. (C) KIAA1429. (D) IGF2BP3. (E) IGF2BP2. (F) EIF3H. (G) Time-dependent ROC analysis of m6Ascore in predicting prognosis. (H) Survival analyses for low and high m6Ascore patient groups using Kaplan-Meier curves.

## Data Availability

The datasets analyzed during the current study are available from TCGA database (https://cancergenome.nih.gov/), GTEx database (https://xenabrowser.net/), Immport database (https://immport.niaid.nih.gov/), TCIA database (https://www.tcia.at/home), GEO database (https://www.ncbi.nlm.nih.gov/geo/), Oncomine database (https://www.oncomine.org).
