# Supplementary material for: Quantification of m6A RNA methylation modulators pattern was a potential biomarker for prognosis and associated with tumor immune microenvironment of pancreatic adenocarcinoma
Source: BMC Cancer. 2021 Jul 31;21:876. doi: 10.1186/s12885-021-08550-9 (PMC8325189; doi:10.1186/s12885-021-08550-9)
Supplement: Supplementary file 2 — Additional file 2: Figure S1. The association between the expression of the modulators and survival time with statistically significant (A) METTL3. (B) IGF2BP3. (C) IGF2BP2. (D) KIAA1429. (E) EIF3H. (F) LRPPRC. Figure S2. Predictive value of m6Ascore in anti-PD-1/L1 immunotherapy based on three immunotherapeutic cohorts. (A) Survival analyses for patients with high or low m6Ascore in GSE78220 cohort. (B) The proportion of patients with response to anti-PD-1/L1 immunotherapy in patients with high or low m6Ascore in GSE78220 cohort. (C) Survival analyses for patients with high or low m6Ascore in TCGA-SKCM cohort (D) The proportion of patients with response to anti-PD-1/L1 immunotherapy in patients with high or low m6Ascore in TCGA-SKCM cohort. (E) Survival analyses for patients with high or low m6Ascore in IMvigor210 cohort (F) The proportion of patients with response to anti-PD-1/L1 immunotherapy in patients with high or low m6Ascore in IMvigor210 cohort. Figure S3. Expression of of six m6A modulators in pancreatic cancer tissues and adjacent normal tissues regarding to oncomine database (A) EIF3H. (B) IGF2BP2. (C) IGF2BP3. (D) KIAA1429. (E) METTL3. (F) LRPPRC. Figure S4. External Validation of six Key Prognostic m6A RNA modulators in GSE62452 dataset. Expression of of six m6A modulators in pancreatic cancer tissues and adjacent normal tissues based on GSE62452 dataset. (A) METTL3. (B) LRPPRC. (C) KIAA1429. (D) IGF2BP3. (E) IGF2BP2. (F) EIF3H. (G) Time-dependent ROC analysis of m6Ascore in predicting prognosis. (H) Survival analyses for low and high m6Ascore patient groups using Kaplan-Meier curves. [file 12885_2021_8550_MOESM2_ESM.docx]

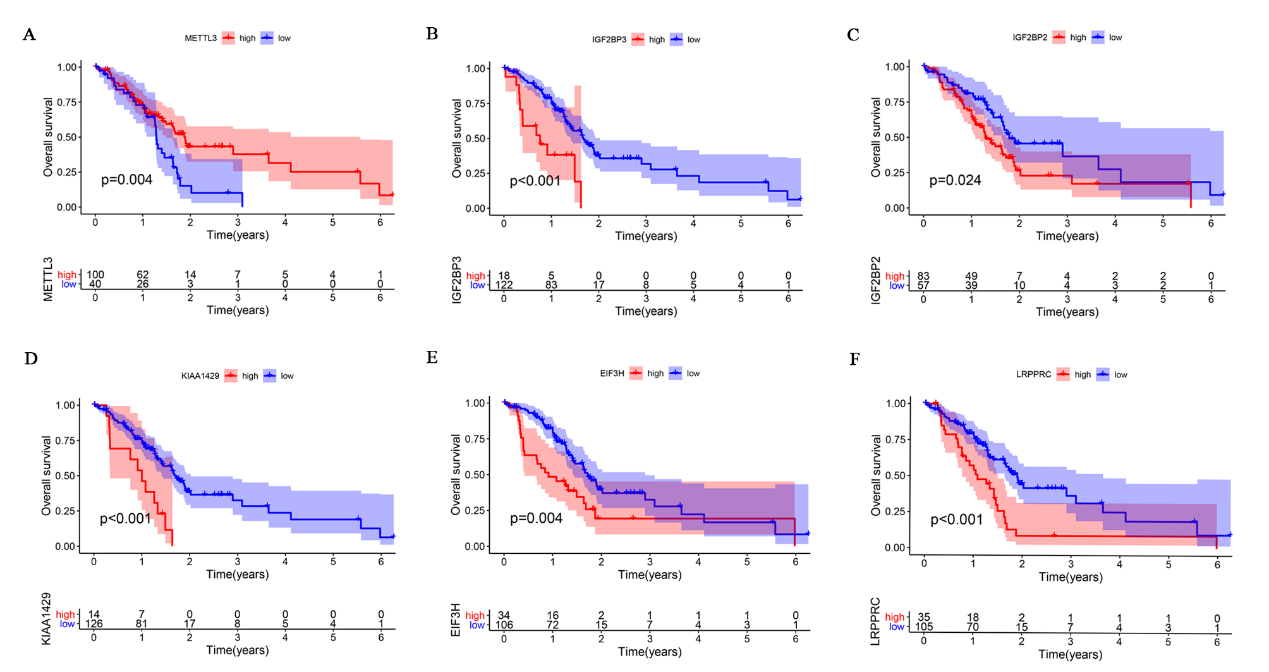


**Figure S1.** The association between the expression of the modulators and survival time with statistically significant (A) METTL3. (B)IGF2BP3. (C) IGF2BP2. (D) KIAA1429. (E) EIF3H. (F)LRPPRC.


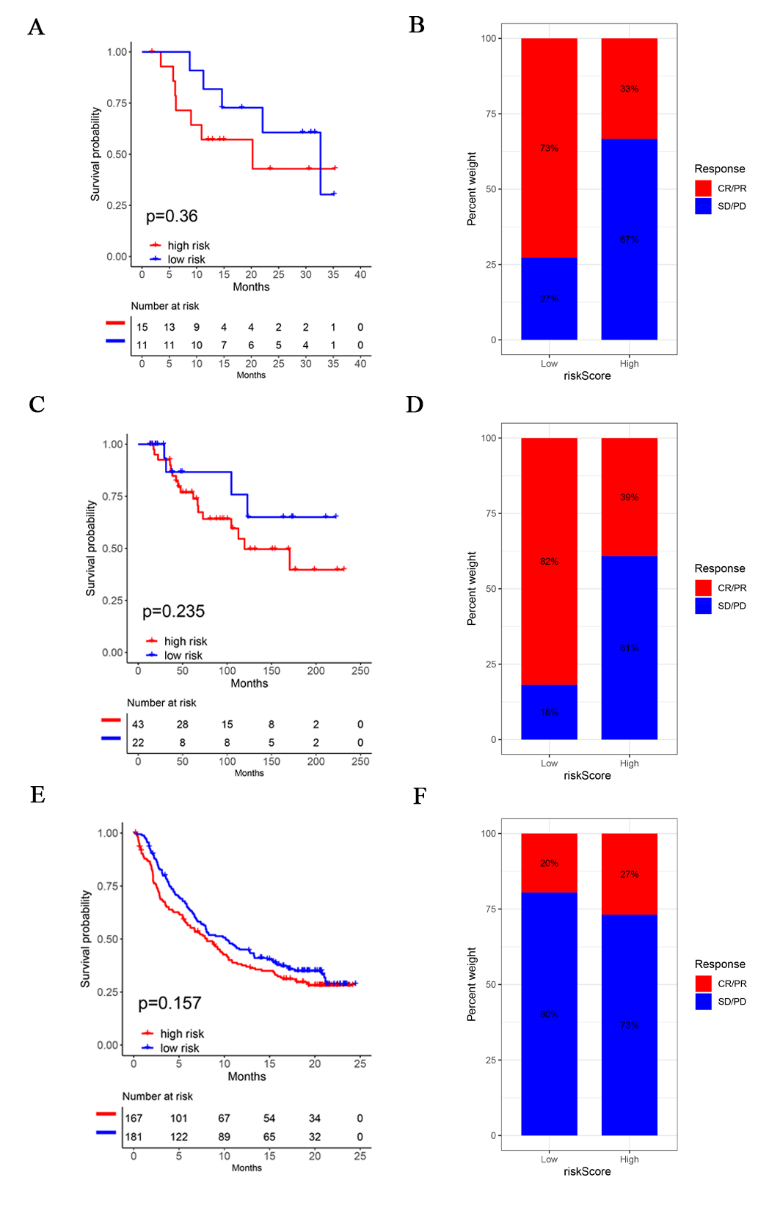


**Figure S2.** Predictive value of m6Ascore in anti-PD-1/L1 immunotherapy based on three immunotherapeutic cohorts. (A)Survival analyses for patients with high or low m6Ascore in GSE78220 cohort. (B) The proportion of patients with response to anti-PD-1/L1 immunotherapy in patients with high or low m6Ascore in GSE78220 cohort. (C)Survival analyses for patients with high or low m6Ascore in TCGA-SKCM cohort (D) The proportion of patients with response to anti-PD-1/L1 immunotherapy in patients with high or low m6Ascore in TCGA-SKCM cohort. (E)Survival analyses for patients with high or low m6Ascore in IMvigor210 cohort (F) The proportion of patients with response to anti-PD-1/L1 immunotherapy in patients with high or low m6Ascore in IMvigor210 cohort.


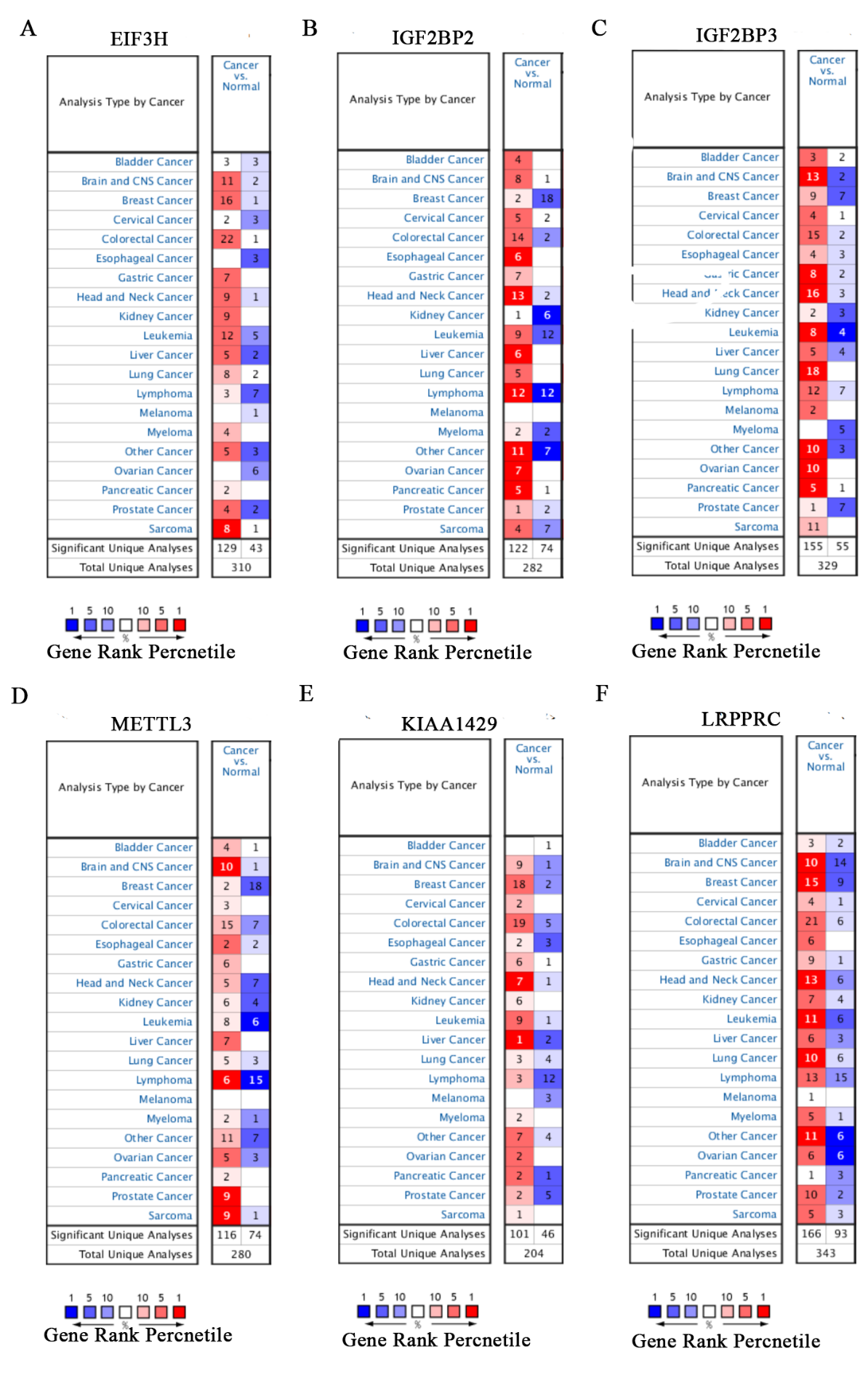


**Figure S3.** Expression of of six m6A modulators in pancreatic cancer tissues and adjacent normal tissues regarding to oncomine database (A) EIF3H. (B) IGF2BP2. (C) IGF2BP3. (D) KIAA1429. (E) METTL3. (F)LRPPRC.


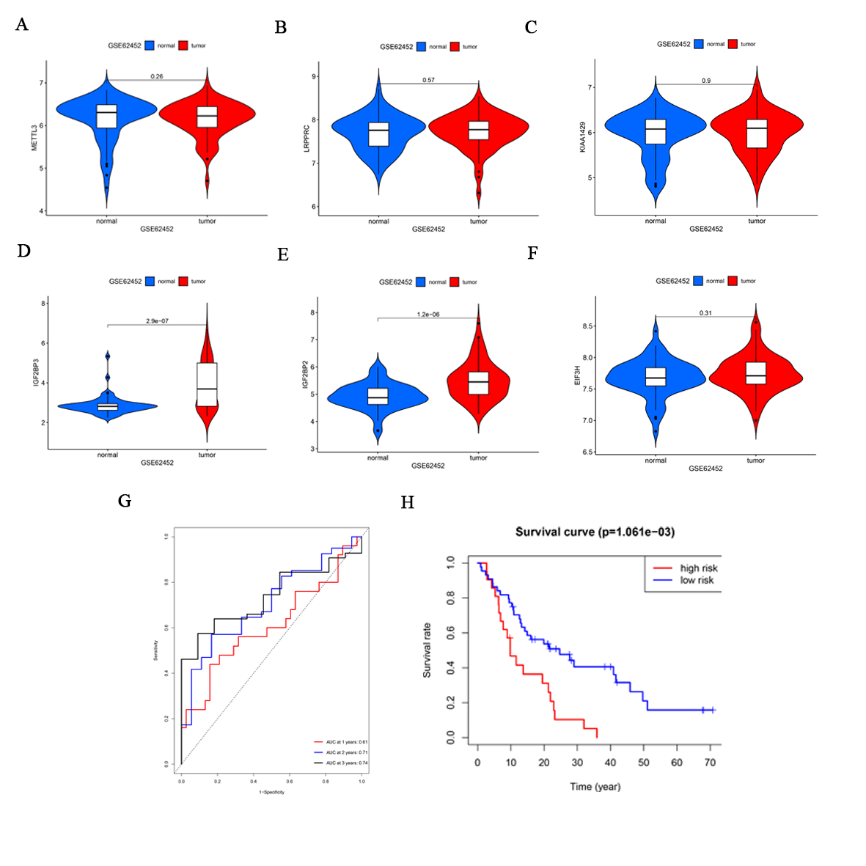


**Figure S4.** External Validation of six Key Prognostic m6A RNA modulators in GSE62452 dataset. Expression of of six m6A modulators in pancreatic cancer tissues and adjacent normal tissues based on GSE62452 dataset. (A) METTL3. (B) LRPPRC. (C) KIAA1429. (D) IGF2BP3. (E) IGF2BP2. (F) EIF3H. (G) Time-dependent ROC analysis of m6Ascore in predicting prognosis. (H) Survival analyses for low and high m6Ascore patient groups using Kaplan-Meier curves.
